# Quelle chimiothérapie dans les tumeurs phyllodes métastatiques du sein? rapport de cas

**DOI:** 10.11604/pamj.2022.42.293.20377

**Published:** 2022-08-18

**Authors:** Imane Ait Kaikai, Mouna Bourhafour, Meriem Haffadi, Zineb Bouchbika, Nadia Benchakroune, Hassan Jouhadi, Nezha Tawfiq, Souha Sahraoui, Abdellatif Benider

**Affiliations:** 1 Centre Mohamed VI pour le Traitement des Cancers, Centre Hospitalier Universitaire Ibn Rochd, Casablanca, Maroc

**Keywords:** Tumeurs phyllodes, sein, métastases, chimiothérapie, cas clinique, Phyllode tumors, breast, metastases, chemotherapy, case report

## Abstract

Les tumeurs phyllodes (TP) du sein sont rares. Elles peuvent revêtir un caractère bénin, borderline ou malin. La forme maligne représente 20-30% des TP avec des métastases à distance dans 10-26% des cas. La chimiothérapie est l´une des principales armes thérapeutiques des tumeurs phyllodes métastatiques (TPM). Nous rapportons quatre cas de TPM du sein prises en charge au centre Mohamed VI de Casablanca pour le traitement des cancers de janvier 2015 à décembre 2017. L´âge de nos patientes était compris entre 25 et 45 ans. Le mode de révélation clinique est représenté, dans la majorité des cas, par l´apparition d´une énorme masse mammaire et le diagnostic histologique a été porté chez toutes les patientes sur la pièce de mastectomie. Trois patientes étaient métastatiques au niveau du poumon, deux avaient des métastases ganglionnaires axillaires, deux avaient des métastases osseuses et une seule avait des métastases hépatiques. Toutes les patientes ont reçu une chimiothérapie. Les drogues utilisées étaient: la doxorubicine en monothérapie et l´association doxorubicine-ifosfamide (AI). Une seule patiente a eu une évolution très favorable avec une réponse radiologique complète après 3 cures d´AI. Les TPM du sein sont de mauvais pronostic. L´intérêt d´une chimiothérapie systémique reste à définir surtout qu´on ne dispose pas jusqu´à présent de données concernant la chimiothérapie optimale.

## Abstract (en)

Phyllodes tumors (PT) of the breast are rare. They can be benign, borderline or malignant. Malignant forms account for 20-30% of PTs, with distant metastases in 10-26% of cases. Chemotherapy is one of the main therapeutic weapons for metastatic phyllodes tumors (MPTs). We here report four cases of MPTs of the breast managed at The Mohammed VI Center For Cancers Treatment in Casablanca from January 2015 to December 2017. The average age of patients ranged from 25 to 45 years. The mode of revelation was represented, in the majority of cases, by the occurrence of a huge breast mass and in all patients the histological diagnosis was based on the examination of mastectomy specimen. Three patients had lung metastases, two had axillary lymph-node metastases, two had bone metastases and only one had liver metastases. All patients received chemotherapy. Doxorubicin monotherapy and doxorubicin-ifosfamide (AI) were used. Only one patient had a very favorable outcome, with radiologic complete response after 3 AI regimens. MPTs of the breast have a poor prognosis. The role of systemic chemotherapy is to be defined, especially since there are no data available on optimal chemotherapy regimen.

## Introduction

Les tumeurs phyllodes (TP) du sein constituent une entité rare. La forme maligne représente moins de 1% de l´ensemble des cancers du sein [1] et peut générer des métastases dans 10-26% des cas [2,3]. La chimiothérapie est l´un des piliers du traitement des tumeurs phyllodes métastatiques (TPM), cependant, celle-ci n´est pas standardisée et son efficacité est inconnue vue la rareté des essais prospectifs randomisés dans ce cadre. Le but de notre étude est de déterminer l´intérêt et le type de chimiothérapie qui paraît optimale dans la prise en charge des TPM tout en soulevant les aspects cliniques, radiologiques, thérapeutiques et évolutives de ce type de tumeurs.

## Patient et observation

### Observation N°1

**Information de la patiente:** femme de 37 ans, traitée en 2002 pour une tumeur phyllode maligne (grade 3) du sein droit, par une tumorectomie sans traitement adjuvant, consulte en 2016 suite à l´apparition d´une masse au niveau du sein homolatéral.

**Résultats cliniques:** la patiente présente un *performans status* à 0, l´examen mammaire objective une masse occupant tout le sein droit mesurant environ 20 cm de grand axe. Il n´y avait pas de masse palpable au niveau du sein controlatéral et les aires ganglionnaires étaient libres (Tableau 1).

**Tableau 1:** synthèse des observations cliniques des patientes

Cas	Age (ans)	Circonstances de découverte	Chirurgie	Délai d´apparition des métastases	Sites de métastases
1	37	Masse du sein droit	Tumorectomie en 2002 mastectomie en 2016	14 ans	Os
2	28	Masse mammaire droite	Tumorectomie à 3 reprises en 2011, 2012 et 2013 mastectomie en 2014	9 mois	Poumon, adénopathies axillaires, adénopathie lombo-aortique
3	25	Autopalpation d´un nodule du sein droit	Mastectomie en 2016	Synchrones	Poumon, ADPs axillaires
4	45	Augmentation progressive du volume du sein gauche	Mastectomie en 2015	Synchrones	Poumon, foie

**Démarche diagnostique:** une mastectomie a été réalisée d´emblée, dont l´étude anatomopathologique a mis en évidence une tumeur phyllode grade 3. Le bilan d´extension fait d´une tomodensitométrie thoraco-abdomino-pelvienne était normal et la patiente n´a reçu aucun traitement adjuvant. Un an plus tard, elle présente une masse dorsale de 5 cm avec une paraplégie. A l´imagerie par résonance magnétique (IRM) médullaire: la masse était en faveur d´une lésion métastatique compliquée d´une épidurite. Une chirurgie décompressive a été réalisée avec, à l´histologie, une prolifération sarcomateuse compatible avec une tumeur phyllode grade 3 infiltrant les parties molles para-vertébrales et les muscles paravertébraux.

**Intervention thérapeutique et suivi:** la patiente a reçu une seule cure de chimiothérapie type doxorubicine en monothérapie vu l´état général limite puis a été perdue de vue (Tableau 2).

**Tableau 2:** protocoles de chimiothérapie reçus et évolution des patientes

Cas	Type de chimiothérapie	Evolution
1	Doxorubicine en monothérapie	Perdue de vue
2	Doxorubicine-ifosfamide (6 cures) ifosfamide en monothérapie (8 cures)	Réponse partielle puis progression après la 8^e^ cure d´ifosfamide
3	Doxorubicine-ifosfamide (3 cures)	Réponse complète
4	Doxorubicine en monothérapie	Décès

**Consentement du patient:** le consentement éclairé de la patiente a été obtenu avant la participation à l´étude.

### Observation N°2

**Information de la patiente:** femme de 28 ans, opérée à 3 reprises pour des nodules du sein droit en 2011, 2012 et 2013. Les examens anatomopathologiques de ces différents nodules n´étaient pas disponibles. Elle consulte en 2014 suite à la réapparition d´une masse siégeant au même sein opéré.

**Résultats cliniques:** la patiente avait un *performans status* à 0, l´examen clinique objectivait une masse mammaire droite mesurant 15 cm de grand axe sans autres lésions ni au niveau du sein controlatéral ni aux aires ganglionnaires (Tableau 1).

**Démarche diagnostique:** elle a bénéficié d´une mastectomie. Une tumeur phyllode maligne a été diagnostiquée histologiquement. Neuf mois plus tard, elle a présenté une récidive locale confirmée par biopsie. Le bilan d´extension fait d´une tomodensitométrie (TDM) thoraco-abdomino-pelvienne a mis en évidence une dizaine de nodules parenchymateux pulmonaires bilatéraux, des adénopathies axillaires droites et une adénopathie lombo-aortique gauche. Ces lésions étaient en faveur de localisations secondaires.

**Intervention thérapeutique et suivi:** la patiente a reçu 6 cures de chimiothérapie type doxorubicine-ifosfamide (AI) avec une réponse radiologique estimée à 30% puis 8 cures d´ifosfamide seul en maintenance. L´évolution a été marquée par l´apparition d´une progression clinico-radiologique marquée par l´altération de l´indice de performance et l´apparition de lésions osseuses secondaires au scanner. Elle a bénéficié d´une radiothérapie antalgique ainsi que de soins de support et est décédée quelques mois après (Tableau 2).

**Consentement du patient:** le consentement éclairé de la patiente a été obtenu avant la participation à l´étude.

### Observation N°3

**Information de la patiente:** femme de 25 ans, ayant dans les antécédents une cholecystectomie, consulte en 2016 suite à l´autopalpation d´un nodule du sein droit.

**Résultats cliniques:** la patiente présentait un *performans status* à 0, l´examen mammaire mettait en évidence une masse mammaire droite mesurant 4 cm sans autres lésions palpables ni au niveau du sein controlatéral ni au niveau des aires ganglionnaires. Aucun autre signe clinique en faveur d´une métastase n´a été noté (Tableau 1).

**Figure 1:**
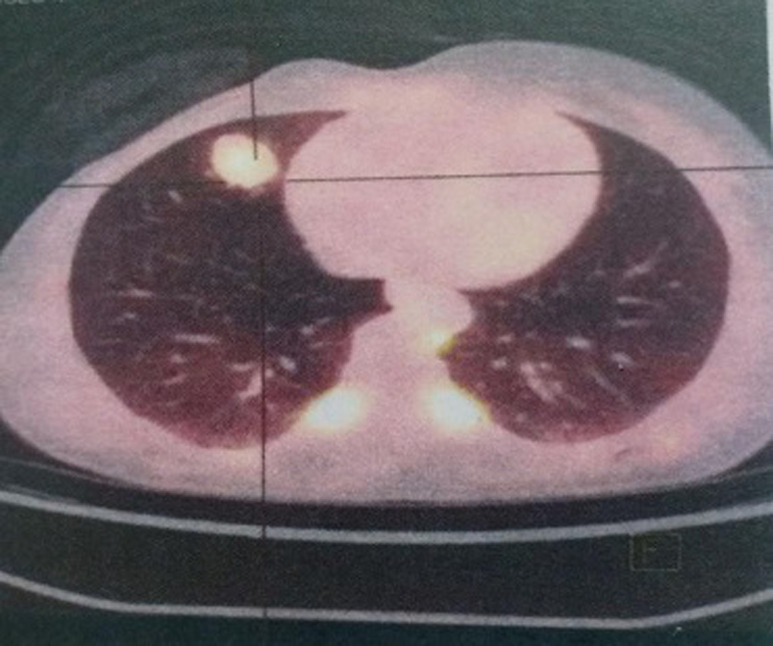
PET scan objectivant un nodule pulmonaire lobaire moyen hypermétabolique en faveur d´une localisation secondaire d´une tumeur phyllode maligne

**Figure 2:**
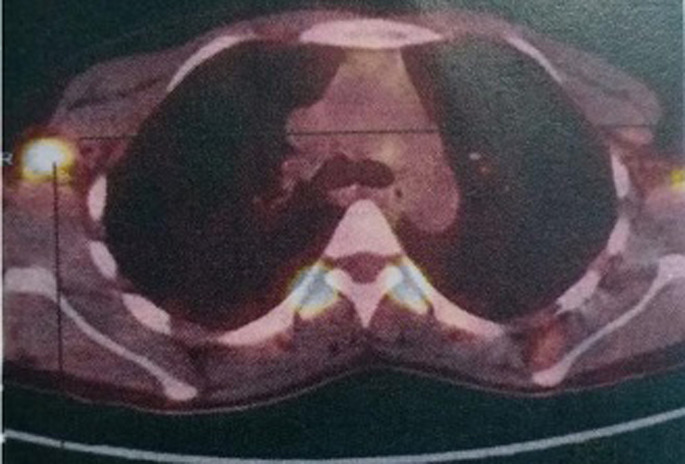
PET scan objectivant une adénopathie axillaire droite métastatique d´une tumeur phyllode maligne du sein

**Démarche diagnostique:** elle a bénéficié d´une biopsie confirmant le diagnostic d´une tumeur phyllode grade 3 puis a eu une mastectomie droite. Dans le cadre du bilan d´extension, la TDM thoraco-abdomino-pelvienne a montré une adénopathie axillaire droite et deux nodules pulmonaires suspects de localisations secondaires. Après la discussion du dossier dans une réunion de concertation pluridisciplinaire (RCP), la décision était de compléter le bilan par une tomographie par émission de positons (PET scan) qui avait objectivé un nodule pulmonaire lobaire moyen hypermétabolique mesurant 23*18 mm d´aspect secondaire (Figure 1) et une adénopathie axillaire droite mesurant 19*11 mm suspecte d´une extension secondaire (Figure 2). Une exérèse de cette adénopathie a été réalisé et l´étude histologique a confirmé sa nature secondaire.

**Intervention thérapeutique et suivi:** la patiente a reçu 3 cycles de chimiothérapie type AI avec une réponse métabolique complète au PET scan d´évaluation. Elle est toujours suivie en consultation avec un bon contrôle radiologique (Tableau 2).

**Consentement du patient:** le consentement éclairé de la patiente a été obtenu avant la participation à l´étude.

### Observation N°4

**Information de la patiente:** femme de 45 ans, ayant dans les antécédents une tumorectomie mammaire droite mettant en évidence une tumeur phyllode grade 1, consulte en 2015 suite à une augmentation progressive du volume du sein opéré.

**Résultats cliniques:** la patiente présente un *performans status* à 0, l´examen mammaire objective une masse occupant tout le sein droit mesurant environ 20 cm de grand axe. Il n´y avait pas de masse palpable au niveau du sein controlatéral et les aires ganglionnaires étaient libres (Tableau 1).

**Figure 3:**
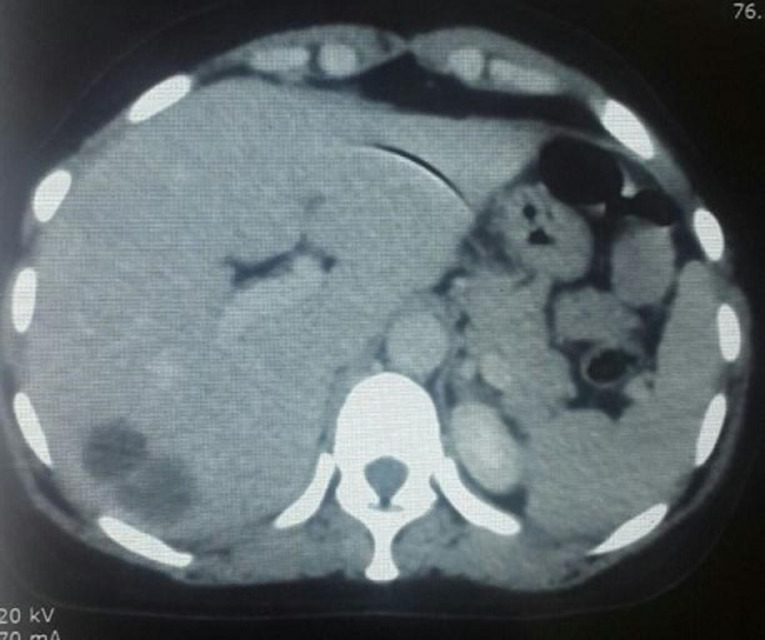
tomodensitométrie (TDM) abdominale montrant une lésion hépatique kystique suspecte d´une métastase d´une tumeur phyllode maligne

**Démarche diagnostique:** une mastectomie droite a été réalisée objectivant une tumeur phyllode grade 3. Le bilan d´extension post-opératoire a montré des lésions kystiques pulmonaires gauches et une lésion hépatique d´allure suspecte (Figure 3). Ces dernières n´étaient pas hypermétaboliques au PET scan. La décision de la RCP était de ne pas considérer la patiente métastatique et de surveiller radiologiquement, d´une façon rapprochée, les lésions hépatiques et pulmonaires.

**Figure 4:**
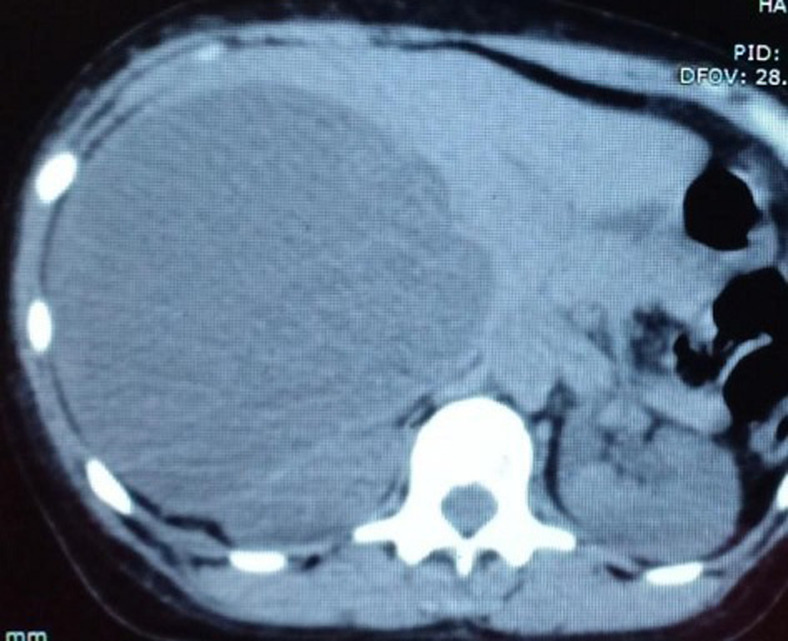
tomodensitométrie (TDM) abdominale d´évaluation objectivant une progression radiologique franche de la lésion hépatique

**Intervention thérapeutique et suivi:** la patiente a reçu une radiothérapie adjuvante. Trois mois plus tard, une augmentation franche de ces lésions a été objectivée sur le scanner d´évaluation (Figure 4). Une chimiothérapie type doxorubicine seule a été proposée, avec une survenue rapide d´une altération de l´état général après une seule cure. Elle est décédée quelques mois après (Tableau 2).

**Consentement du patient:** le consentement éclairé de la patiente a été obtenu avant la participation à l´étude.

## Discussion

Découvertes en 1838 par Müller [4], les TP du sein sont rares. Elles représentent des néoplasies fibro-épithéliales constituées à la fois d´un contingent épithélial et d´un stroma hypercellulaire. Selon l´organisation mondiale de la santé (OMS), les TP sont classés en tumeurs bénignes, borderlines et malignes en évaluant cinq paramètres histologiques: la cellularité stromale, l´atypie stromale, les mitoses, la prolifération stromale et les marges de la tumeur. La forme maligne est dotée d´un potentiel métastatique à distance, essentiellement par voie hématogène. Ces métastases peuvent être découvertes d´une façon synchrone ou métachrone, leur délai d´apparition varie selon les différentes études entre 7 mois et 6 ans après la chirurgie [5]. Dans notre série, 2 patientes avaient des métastases synchrones et 2 patientes avaient des métastases métachrones ayant survenu 9 mois et 14 ans après la chirurgie initiale. Le poumon et l´os représentent les sites de prédilection des métastases des TP [2]. Ce constat rejoint les résultats de notre étude puisque le poumon occupait la première position des sites métastatiques suivi de l´os puis des adénopathies axillaires. D´autres localisations ont été retrouvées chez nos patientes, à savoir le foie et les ganglions rétropéritonéales.

Si une chirurgie optimale basée sur une tumorectomie élargie ou une mastectomie sans curage ganglionnaire constitue le traitement de base des formes localisées, la chimiothérapie est considérée comme le pilier du traitement des TPM. Plusieurs protocoles de chimiothérapie ont été étudiés dans la littérature, à base de mono ou polychimiothérapie. Dans ce sens, Burton *et al*. ont traité, en 1982, 3 patientes ayant des TPM par une chimiothérapie à base d´étoposide cisplatine; un bénéfice clinique et une réponse radiologique partielle ont été notés chez 2 patientes [6]. Une étude belge ayant comporté un seul cas de TPM traité par le même protocole a mis en évidence aussi une rémission partielle après six cycles de chimiothérapie [7].

À travers une série plus large de 37 patients réalisée par Mitus *et al*., une monochimiothérapie par cyclophosphamide ou ifosfamide n´a pas prouvé une certaine efficacité avec des médianes de survie faibles de 3 et 5 mois respectivement [8]. La même étude a objectivé un allongement de la survie chez les patients recevant des protocoles de chimiothérapie à base de doxorubicine seule (7 mois) ou en association avec cisplatine, cyclophosphamide ou ifosfamide (9 mois). En analysant les patients en sous-groupes, Mitus *et al*. ont objectivé une meilleure survie arrivant à 11,8 mois chez les patients ayant uniquement des métastases osseuses et traitées par doxorubicine associée à une radiothérapie palliative; les patients ayant des métastases cérébrales et traités également par une radiothérapie palliative associée à une chimiothérapie par doxorubicine seul, avaient le taux de survie le plus faibles (2,8 mois). Chez les patients présentant uniquement des métastases pulmonaires et qui constituaient la majorité des cas de cette série rétrospective, la survie variait entre 3 et 11 mois; une rémission complète a été notée, notamment chez ceux ayant bénéficié d´une résection des lésions pulmonaires associée à la doxorobucine en monothérapie (2 patients) ou traités par doxorubicine cisplatine (1 patient).

D´une façon générale, les médianes de survie les plus élevées étaient observées chez les patients traités par polychimiothérapie à base de doxorubicine [8], ce qui rejoint les résultats de notre série de cas où une chimiothérapie de type doxorubicine ifosfamide associée à une résection des métastases ganglionnaires axillaires a permis d´obtenir, chez une seule malade, une rémission complète après 3 cures. Cette dernière est toujours suivie avec un bon contrôle radiologique à 2 ans de la fin du traitement. Le même protocole a permis une rémission partielle chez une autre malade qui n'a duré que quelques mois. Cela nous mène à suggérer que la chirurgie des métastases, si celles-ci étaient accessibles ou la radiothérapie, pourraient potentialiser le rôle de la chimiothérapie et améliorer les taux de survie.

D´autre part, Kan Yonemoria *et al*. ont traité 8 patientes par une chimiothérapie intensifiée comportant doxorubicine, dacarbazine, ifosfamide et mesna. Une réponse partielle a été mise en évidence chez 4 patientes au prix d´une toxicité hématologique de grade 3 avec une médiane de survie de 5 mois [9]. À travers les différentes études citées, on constate qu´un régime de chimiothérapie muli-agent à base de doxorubicine est le plus souvent utilisé avec une efficacité qui paraît légèrement supérieure de la polychimiothérapie par rapport à la monochimiothérapie. Mais, malgré un taux de réponse observé par ces drogues, le protocole de chimiothérapie optimal n´est pas encore identifié et l´efficacité de la chimiothérapie reste controversée.

## Conclusion

Les tumeurs phyllodes métastatiques du sein sont rares. Leur prise en charge systémique ne relève pas d´un consensus standard. L´efficacité d´une chimiothérapie systémique reste difficile à prouver vue la rareté de ce type de tumeurs et le nombre limité de séries de cas étudiés. L´étude des marqueurs qui permettront de sélectionner les patients qui répondront le mieux à la chimiothérapie, ainsi que le rôle d´autres traitements tels que la chirurgie des métastases et la radiothérapie dans la potentialisation de l´efficacité de la chimiothérapie, devront être précisés dans le futur par des études plus larges.
